# MGMT Leu84Phe Polymorphism Contributes to Cancer Susceptibility: Evidence from 44 Case-Control Studies

**DOI:** 10.1371/journal.pone.0075367

**Published:** 2013-09-26

**Authors:** Jun Liu, Renxia Zhang, Fei Chen, Cuicui Yu, Yan Sun, Chuanliang Jia, Lijing Zhang, Taufiq Salahuddin, Xiaodong Li, Juntian Lang, Xicheng Song

**Affiliations:** 1 Department of Otolaryngology Head and Neck Surgery, West China Hospital, Sichuan University, Chengdu, Sichuan, China; 2 Department of Anesthesia, Yuhuangding Hospital, Medical School of Qingdao University, Yantai, Shandong, China; 3 Department of Otolaryngology Head and Neck Surgery, Yuhuangding Hospital, Medical School of Qingdao University, Yantai, Shandong, China; 4 Binzhou Medical School, Yantai, Shandong, China; 5 Qingdao Medical School, Qingdao, Shandong, China; 6 Wake Forest School of Medicine, Winston-Salem, North Carolina, United States of America; 7 The 3^rd^People’s Hospital of Jinan, Jinan, Shandong, China; 8 Department of Otolaryngology Head and Neck Surgery, Shanghai Changzheng Hospital, Second Military Medical University, Shanghai, China; San Francisco Coordinating Center, United States of America

## Abstract

**Background:**

O^6^-methylguanine-DNA methyltransferase is one of the few proteins to directly remove alkylating agents in the human DNA direct reversal repair pathway. A large number of case-control studies have been conducted to explore the association between MGMT Leu84Phe polymorphism and cancer risk. However, the results were not consistent.

**Methods:**

We carried out a meta-analysis of 44 case-control studies to clarify the association between the Leu84Phe polymorphism and cancer risk.

**Results:**

Overall, significant association of the T allele with cancer susceptibility was verified with meta-analysis under a recessive genetic model (*P*<0.001, OR=1.30, 95%CI 1.24-1.50) and TT versus CC comparison (*P*=0.001, OR=1.29, 95% CI 1.12-1.50). In subgroup analysis, a significant increased risk was found for lung cancer (TT versus CC, P=0.027, OR=1.67, 95% CI 1.06-2.63; recessive genetic model, *P*=0.32, OR=1.64, 95% CI 1.04-2.58), whereas risk of colorectal cancer was significantly low under a dominant genetic model (*P*=0.019, OR=0.84, 95% CI 0.72-0.97). Additionally, a significant association between TT genetic model and total cancer risk was found in the Caucasian population (TT versus CC, P=0.014, OR=1.29, 95% CI 1.05-1.59; recessive genetic model, P=0.009, OR=1.31, 95% CI 1.07-1.61), but not in the Asian population. An increased risk for lung cancer was also verified in the Caucasian population (TT versus CC, P=0.035, OR=1.62, 95% CI 1.04-2.53; recessive genetic model, *P*=0.048, OR=1.57, 95% CI 1.01-2.45).

**Conclusions:**

These results suggest that MGMT Leu84Phe polymorphism might contribute to the susceptibility of certain cancers.

## Introduction

Over the past decades, there has been an increasing understanding of the disease process in human carcinoma. It is now well established that carcinoma can be initiated by DNA damage from UV exposure, ionizing radiation, environmental chemical agents, and byproducts of cell metabolism. Normally, when DNA damage occurs, DNA repair systems recognize the DNA lesions, excise them, and restore the DNA to maintain genome stability and integrity [1]. However, if genetic alterations occur in genes encoding DNA repair proteins, the DNA repair process may be impaired, potentially contributing to an increased risk for developing cancers.

The O^6^-methylguanine-DNA methyltransferase (MGMT) is one of the most important proteins in the DNA repair process. It is a 207 amino acid zinc-bound protein which is encoded by MGMT gene located on chromosome 10 at 10q26 and spans approximately 300kb [2]. It has been shown that MGMT has basic methyl-transferring activity [3] and plays a central role in the cellular defense against alkylating agents within the human DNA direct reversal repair pathway.

Also known as O^6^-alkylguanine–DNA alkyltransferase (ATase, AGT, or AGAT), MGMT protein can directly remove alkyl or methyl adducts from the *O* ^*6*^position of guanine to an internal cysteine residue at codon 145 of the protein [4]. By which, it protects cells against potential DNA alkylation damage from endogenous and exogenous alkylating species such as cigarette consumption, environmental contaminants, and diet [5]. Additionally, it seems that MGMT lacks the ability to dealkylate itself. MGMT therefore can take part only in a single reaction, in which it is irreversibly inactivated [6]. Hence, the reaction should be stoichiometric rather than catalytic. The MGMT expression shows significant variation not only among different body tissues [7], but also among individuals in the same specific tissue [8]. Though the causes of the inter-individual differences in MGMT protein expression levels remain unclear to date, functional polymorphisms in the MGMT gene may have the potential to affect DNA repair capacity. Because of its important role in human DNA direct reversal repair pathway, MGMT has attracted significant attention as a candidate susceptibility gene for cancer.

A large number of molecular epidemiology studies have been carried out to assess the roles of the MGMT polymorphisms in various types of cancer, including lung cancer, head and neck cancer, and colorectal cancer [9,10,11,12,13,14,15,16,17,18,19,20,21]. The MGMTLeu84Phe substitution is the most widely studied polymorphism in MGMT due to a (C->T) transition at nt.262 (MGMT Leu84Phe, rs12917). However, numerous studies on the association of the MGMT Leu84Phe polymorphism with cancer risk have yielded inconsistent results and even partially contradictory conclusions. Several factors may contribute to the discrepancies among different studies. The differences of tumor sites, ethnicities or sample size may all cause the bias of the result of each individual study.

Since single studies may have been underpowered in clarifying the associations of MGMT polymorphisms with cancer susceptibility, to address the controversy among literatures, in the present study we conducted an evidence-based quantitative meta-analysis of the association between the *MGMT* Leu84Phe polymorphism and susceptibility to cancer.

## Materials and Methods

### Identification and eligibility of relevant studies

To identify all studies that explored the association of MGMT Leu84Phe polymorphism with cancer risk, we carried out a computerized literature search of the PubMed database (up to July 20, 2012), using the following key words: ‘MGMT,’ ‘polymorphism,’ and ‘cancer,’ without any restriction on language or publication year. The searched papers were read and assessed for their appropriateness of including. All references cited in the articles were also read to identify relevant publications. Eligible studies should meet two criteria: (1) case-control studies; and (2) genotype frequencies in both cancer cases and controls were available. Exclusion criteria were as follows: (a) not relevant to MGMT Leu84Phe polymorphism; (b) not case-control study; (c) control population included malignant tumor cases; and (d) article was a review or duplication of previous publication.

### Data extraction

The data was extracted by two investigators (Jun Liu and Fei Chen) from each article independently. Discrepancies were not solved until consensus was reached on every item. From each study, the following data were collected: author’s name, year of publication, country of origin, racial descent, cancer type, source of the control population, genotyping methods, matched factors as well as adjusted factors, number of cases and controls, genotype frequencies for cases and controls, characteristics of cancer cases, and controls. If data of subpopulation from different ethnicities was available in one paper, we took each subpopulation as an individual study.

### Statistical analysis

Hardy-Weinberg equilibrium (HWE) for each study was assessed using goodness-of-fit test (x^2^ of Fisher’s exact test) only in control groups [22]. Crude odds ratios (ORs) with 95% confidence intervals (CIs) were calculated to evaluate the strength of association between MGMTLeu84Phe polymorphism and cancer susceptibility. In the overall and subgroup meta-analysis, we evaluated the associations of genetic variants with cancer risk in homozygous genetic contrast (TT vs. CC), dominant geneticmodel (CT+TT vs. CC), recessive genetic model (TT vs. CT+CC) and T allele vs C allele. The significance of the pooled OR was assessed by the Z-test (*P*<0.05 shows a significant association). In addition to overall meta-analysis, stratified analysis on ethnicity (Asians, Caucasians, and the other ethnicities group) and tumor site was also performed A x^2^-based Q-test was carried out to assess the heterogeneity of the ORs [23]. If the result of heterogeneity test was *P*>0.1, ORs were pooled according to the fixed-effects model (Mantel-Haenszel model). Otherwise, the random-effects model (DerSimonian and Laird model) was applied [24]. The Egger regression test and Begg-Mazumdar test were utilized to measure the potential publication bias [25]. All statistical tests were conducted with the software STATA v.10.0 (Stata Corporation, College Station, TX, USA) using two-side *P* values.

## Results

### Characteristics of studies

**Figure 1:**
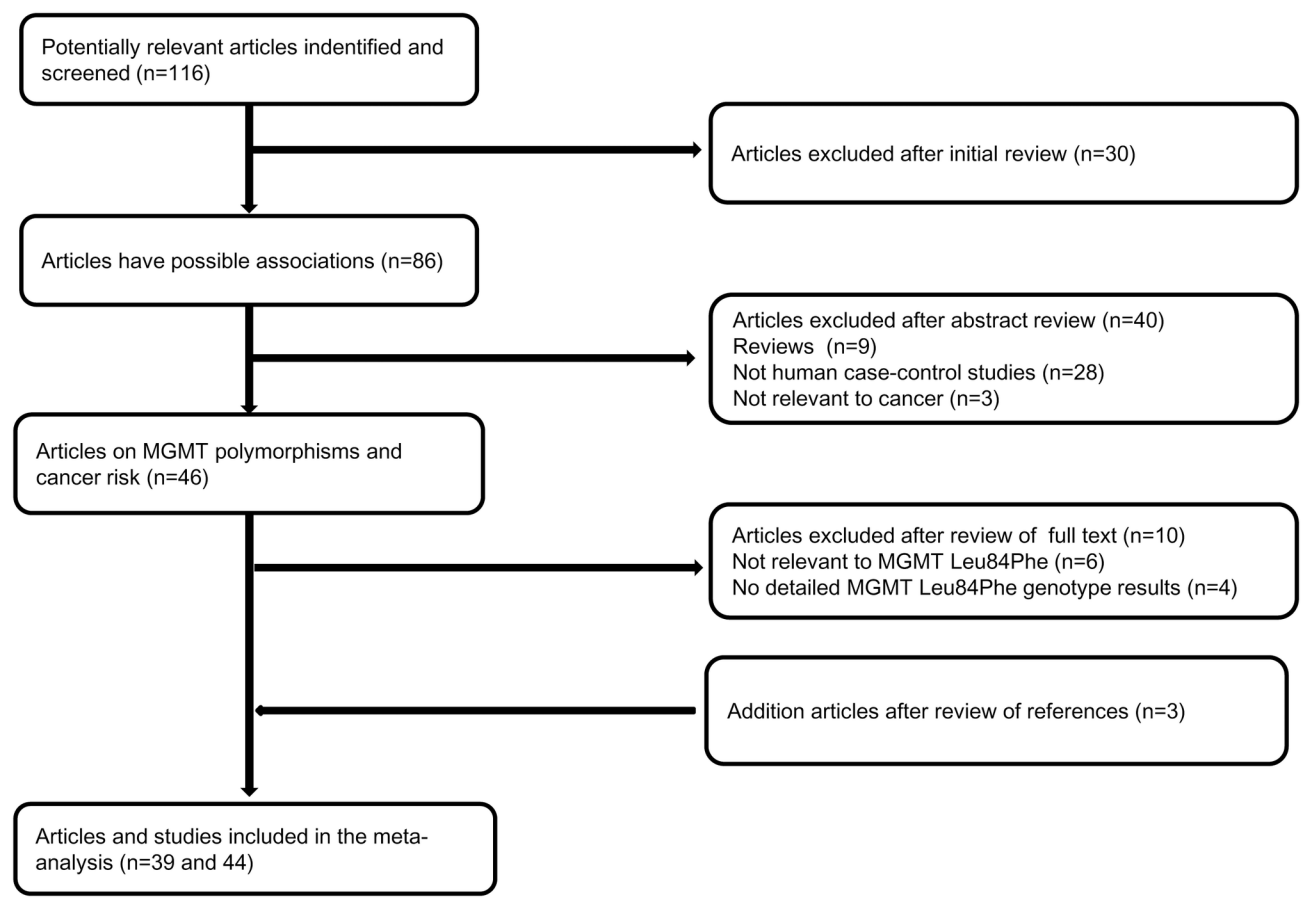
Studies identified with criteria for inclusion and exclusion.

The preliminary literature search yielded 46 articles that explored the association of MGMT polymorphisms with the susceptibility to different cancers. However, six articles [26,27,28,29,30,31] irrelevant to MGMT Leu84Phe polymorphism and four articles [32,33,34,35] without detailed MGMT Leu84Phe genotypes data were excluded. In addition, three articles [10,36,37] were included by literature reading and manual searching. Therefore, 39 articles [9-21, 36-61] were identified and included in the final meta-analysis (Figure **1**). Five papers [14], [18] [56], [59], and [61] presented data including more than one racial populations and each subgroup in these studies was taken as a separate study. Therefore, a total of 44 studies from 39 papers (18938 cancer patients and 28796 controls) were included. All of the cases were confirmed by histological or pathological examination. A classic polymerase chain reaction-restriction fragment length polymorphism (PCR-RFLP) assay was adopted only in 7 of 44 studies and some other genotyping methods were also used widely, such as Taqman, sequencing and Illumina SNP genotyping BeadLab platform. All the genotyping methods are valid for the present meta-analysis. All studies stated that the gender status and the age range were matched between case and control population. The characteristics of included studies are listed in Table **1**. All studies were case-control studies or nested case-control studies within prospective cohort studies, including 9 upper aerodigestive tract squamous cell carcinoma (UADT SCC) studies, 7 colorectal cancer studies, 5 lung cancer studies, 4 brain cancer studies, 3 prostate studies and 13 studies on “other cancers”. There were 15 studies of Caucasian ethnicity, 13 studies of Asian ethnicity, and 16 studies of “mixed ethnicities” (including studies of American, Australian, Black and unspecified population, which cannot be categorized as a unique group since it is mixed). The detailed MGMT Leu84Phe genotype distributions and allele frequencies for cancer cases and controls were presented in Table **2**. The equilibrium of genotypes in the controls was consistent with HWE in all but five studies [9,10,17,21,45] (*P*=0.01, *P*=0.06, *P*=0.02, *P*<0.01, *P*=0.04, respectively) (Table **2**).

**Table 1:** Characteristics of studies included in the meta-analysis.

First author and published year	Country	Cancer	Racial descent	Source of controls	No. of cases/controls	Matching
Inoue (2003)	Japan	Brain tumors	Asian	Population	73/224	Age
Krzensniak (2004)	Poland	Lung cancer	Caucasian	Population	96/96	Age,Sex,Smoking
Bigler (2005)	America	Colorectal cancer	American	Hospital	517/615	None
Huang (2005)	Poland	Gastric cancer	Caucasian	Population	280/387	Age,Sex
Huang (2005) 1	America	Head and neck SCC	Caucasian	Population/hospital	400/665	Age,Sex, Race
Huang (2005) 2	America	Head and neck SCC	Non-white American	Population/hospital	114/89	Age,Sex, Race
Li (2005)	China	Bladder cancer	Asian	Population	167/204	Age,Sex, Smoking
Ritchey (2005)	China	Prostate cancer	Asian	Population	161/246	Age
Shen (2005)	America	Breast cancer	American	Population	1064/1107	Age
Chae (2006)	Korea	Lung cancer	Asian	Hospital	432/432	Age,Sex
Han (2006)	America	Endometrial cancer	Caucasian	Population	434/1085	Age
Han (2006)	America	Breast cancer	Caucasian	Population	1276/1714	Age
Jiao (2006)	America	Pancreatic cancer	American	Hospital	370/340	Age,Sex, Race
Kietthubthew (2006)	Thailand	Oral SCC	Asian	Population	106/164	Age,Sex
Moreno (2006)	Spain	Colorectal cancer	Caucasian	Hospital	272/299	None
Tranah (2006) 1	America	Colorectal cancer	American (PHS)^c^	Hospital	186/2137	Age,Smoking
Tranah (2006) 2	America	Colorectal cancer	American (NHS)^d^	Hospital	257/429	Age
Wang (2006)	America	Lung cancer	Caucasian	Hospital	1121/1163	Age,Sex, Race,Smoking,
Zienolddiny (2006)	Norway	Lung cancer	Caucasian	Population	304/363	Age,Smoking
Felini (2007)	America	Gliomas	American	Population	379/459	Age,Sex, Race
Hall (2007)	Europe^a^	UADT SCC	Caucasian	Hospital	803/1062	Age,Sex, Residence
Hu (2007)	China	Lung cancer	Asian	Hospital	500/517	Age,Sex, residence
Huang (2007)	China	Cervical cancer	Asian	Hospital	539/800	Age,Residence
Shen (2007)	Australia	Non-Hodgkin’s lymphoma	Australian	Population	555/495	Age,Sex, Residence
Stern (2007)	Singapore	Colorectal cancer	Asian	Population	292/1166	None
Doecke (2008)	Australia	Esophageal adenocarcinoma	Australian	Population	566/1337	Age,Residence
Zhang (2008)	China	Biliary tract cancer	Asian	Population	406/782	None
Hazra (2008)	America	Colorectal cancer	American	Population	358/357	Age
kbari (2009)	Iran	Esophageal SCC	Asian	Hospital	196/250	None
Gu (2009)	America	Melanoma	American	Population	214/212	Age, Race
Khatami (2009)	Iran	Colorectal cancer	Asian	Hospital	200/201	Age,Sex
Liu (2009)	America	Glioma	American	Population	369/363	Age,Sex, Race
McKean-Cowdin (2009)	America	Glioblastoma	Caucasian	Population/hospital	998/1968	Age,Sex, Race
Yang (2009)	China	Non-Hodgkin’s lymphoma	Asian	Hospital	48/352	None
Agalliu (2010) 1	America	Prostate cancer	Caucasian	Population	1250/1237	Age
Agalliu (2010) 2	America	Prostate cancer	African-American	Population	147/81	Age
Huang (2010)	America	Oral SCC	Asian	Hospital	176/110	None
Palli (2010)	China	Gastric cancer	Caucasian	Population	291/537	None
Zhang (2010)	Italy	Head and neck SCC	Caucasian	Hospital	721/1234	Age,Sex
Bye (2011) 1	America	Esophageal SCC	Black	Population	346/469	Age,Sex, Race
Bye (2011) 2	South Africa	Esophageal SCC	Mixed ethnicities	Population	196/423	Age,Sex, Race
Loh (2011)	South Africa	Cancers	Caucasian	Population	188/1120	None
O’Mara (2011) 1	UK^b^	Endometrial cancer	Australian	Population	1173/1099	Age,Residence
O’Mara (2011) 2	Australia	Endometrial cancer	Caucasian	Population	397/406	Age

SCC- squamous cell carcinoma；UADT SCC - Upper Aerodigestive Tract Squamous Cell Carcinoma

a: Include 5 central and eastern European countries

b: Indlude Norfolk, East Anglia and United Kingdom

c: PHS- Physicians’ Health Study d: NHS-Nurses’ Health Study

**Table 2:** Distribution of MGMT Leu84Phe genotypes and allelic frequency.

Study (year)	Distribution of MGMT Leu85Phe genotypes								Frequency of MGMT Leu85Phe alleles					HWE *P* value
	Case (n)				Control (n)				Case (n)			Control (n)		
	CC	CT	TT		CC	CT	TT		C	T		C	T	
Inoue (2003)	55	18	0		160	55	9		128	18		375	73	0.13
Krzensniak (2004)	67	23	6		74	17	5		157	35		165	27	**0.01**
Bigler (2005)	403	108	6		466	136	13		914	120		1068	162	0.41
Huang (2005)	190	82	8		279	99	9		462	98		657	117	0.95
Huang (2005) a	315	80	5		468	179	18		710	90		1115	215	0.86
Huang (2005) b	71	37	6		61	25	3		179	49		147	31	0.82
Li (2005)	132	34	1		173	28	3		298	36		374	34	0.15
Ritchey (2005)	123	36	2		213	32	1		282	40		458	34	0.86
Shen (2005)	778	265	21		824	263	20		1821	307		1911	303	0.85
Chae (2006)	344	84	4		341	81	10		772	92		763	101	**0.06**
Han (2006)	344	82	8		822	242	21		770	98		1886	284	0.52
Han (2006)	964	279	33		1306	382	26		2207	345		2994	434	0.75
Jiao (2006)	264	101	5		257	82	1		629	111		596	84	**0.04**
Kietthubthew (2006)	84	21	1		130	33	1		189	23		293	35	0.48
Moreno (2006)	213	47	12		225	63	11		473	71		513	85	**0.02**
Tranah (2006) a	147	33	6		1634	471	32		327	45		3739	535	0.77
Tranah (2006) b	204	47	6		330	93	6		455	59		753	105	0.85
Wang (2006)	832	259	30		872	272	19		1923	319		2016	310	0.67
Zienolddiny (2006)	189	102	13		247	106	10		480	128		600	126	0.73
Felini (2007)	289	84	6		369	84	6		662	96		822	96	0.63
Hall (2007)	574	198	31		764	277	21		1346	260		1805	319	0.48
Hu (2007)	418	77	5		421	93	3		913	87		935	99	0.38
Huang (2007)	372	156	11		592	198	10		900	178		1382	218	0.15
Shen (2007)	432	112	11		373	110	12		976	134		856	134	0.26
Stern (2007)	251	40	1		959	194	13		542	42		2112	220	0.37
Doecke (2008)	416	136	14		1029	281	27		968	164		2339	335	0.13
Zhang (2008)	352	53	1		631	144	7		757	55		1406	158	0.70
Hazra (2008)	271	72	15		254	97	6		614	102		605	109	0.34
Akbari (2009)	142	53	1		185	63	2		337	55		433	67	0.17
Gu (2009)	152	60	2		168	43	1		364	64		379	45	0.32
Khatami (2009)	40	160	0		61	140	0		240	160		262	140	**0.00**
Liu (2009)	299	62	8		267	89	7		660	78		623	103	0.89
McKean-Cowdin (2009)	774	204	20		1480	453	35		1752	244		3413	523	0.96
Yang (2009)	33	14	1		289	58	5		80	16		636	68	0.29
Agalliu (2010) a	949	269	32		916	298	23		2167	333		2130	344	0.83
Agalliu (2010) b	106	35	6		60	20	1		247	47		140	22	0.64
Huang (2010)	151	25	0		89	21	0		327	25		199	21	0.27
Palli (2010)	210	77	4		395	131	11		497	85		921	153	0.97
Zhang (2010)	563	151	7		933	284	17		1277	165		2150	318	0.38
Bye (2011) a	225	111	10		300	155	14		561	131		755	183	0.26
Bye (2011) b	120	65	11		294	116	13		305	87		704	142	0.71
Loh (2011)	146	37	5		894	212	14		329	47		2000	240	0.72
O’Mara (2011) a	889	261	23		810	270	19		2039	307		1890	308	0.52
O’Mara (2011) b	278	108	11		296	103	7		664	130		695	117	0.57

Bold indicates statistically significant *P* value.

HWE Hardy–Weinberg equilibrium

### Quantitative synthesis

In overall analysis, significant associations between the T allele and cancer risk were found under the recessive genetic model (*P*=0.001, OR=1.28, 95%CI 1.11-1.47) and TT versus CC comparison (*P*=0.001, OR=1.28, 95% CI 1.11-1.47). And, after we excluded those studies whose genotype equilibrium was not consistent with HWE, significant associations between the T allele and cancer susceptibility was also uncovered under the recessive genetic model (*P*<0.001, OR=1.30, 95%CI 1.24-1.50) and TT versus CC comparison (*P*=0.001, OR=1.29, 95% CI 1.12-1.50). However, no significant association was found in the dominant genetic model (TT+TC versus CC) and T versus C comparison. These results were summarized in Table **3**.

**Table 3:** Summary ORs (95% CI) for MGMT Leu84Phe variant under different genetic models and tumor site.

MGMT Leu85Phe	N^#^	TT versus CC			CT+TTversus CC			TT versus CT+CC			T versus C	
					(dominant genetic model)			(recessive genetic model)				
Tumor site		OR (95%CI)	*P*		OR (95%CI)	*P*		OR (95%CI)	*P*		OR (95%CI)	*P*
Total	44	1.28 (1.11-1.47)	**0.001**		1.01 (0.94-1.08)^b^	0.808		1.28 (1.11-1.47)	**0.001**		1.01 (0.96-1.08)^b^	0.504
Total in HWE	39	1.29 (1.12-1.50)	**0.001**		1.00 (0.93-1.07)^b^	0.890		1.30 (1.24-1.50)	**0.000**		1.01 (0.95-1.08)^b^	0.692
UADT SCC	9	1.24 (0.89-1.73)	0.197		0.96 (0.82-1.13)^b^	0.626		1.25 (0.90-1.73)	0.189		0.98 (0.84-1.15)^b^	0.820
Colorectal cancer	7	1.29 (0.85-1.95)	0.234		0.89 (0.78-1.02)	0.091		1.35 (0.90-2.04)	0.152		0.94 (0.84-1.05)	0.267
Colorectal cancer in HWE	5	1.25 (0.62-2.50)^b^	0.536		0.84 (0.72-0.97)	**0.019**		1.30 (0.64-2.66)^b^	0.470		0.88 (0.77-1.01)	0.073
Lung cancer	5	1.38 (0.92-2.06)	0.119		1.05 (0.92-1.19)	0.485		1.34 (0.90-2.00)	0.147		1.06 (0.95-1.20)	0.298
Lung cancer in HWE	3	1.67 (1.06-2.63)	**0.027**		1.05 (0.91-1.21)	0.526		1.64 (1.04-2.58)	**0.032**		1.08 (0.95-1.23)	0.232
Brain cancer	4	1.11 (0.71-1.73)	0.664		0.89 (0.68-1.16)^b^	0.390		1.42 (0.73-1.79)	0.562		0.90 (0.72-1.13)^b^	0.375
Prostate cancer	3	1.48 (0.88-2.48)	0.136		1.22 (0.74-2.00)^b^	0.445		1.51 (0.91-2.53)	0.113		1.25 (0.81-1.94)^b^	0.321
Endomtrial cancer	3	1.14 (0.74-1.77)	0.560		0.92 (0.80-1.06)	0.240		1.64 (0.75-1.80)	0.495		0.95 (0.84-1.07)	0.394
Other cancers	13	1.17 (0.88-1.54)	0.281		1.10 (0.97-1.26)^b^	0.147		1.14 (0.87-1.51)	0.350		1.09 (0.97-1.23)^b^	0.152
Other cancers in HWE	12	1.14 (0.86-1.51)	0.368		1.09 (0.95-1.26)_b_	0.216		1.12 (0.84-1.47)	0.446		1.08 (0.95-1.22)^b^	0.236

Bold indicates statistically significant *P* value

All summary ORs were calculated using fixed-effects models, unless stated otherwise

# Number of studies

b Random-effect models

HWE − Hardy Weinberg Equilibrium

When the subgroup analyses were carried out according to tumor site, the MGMT T allele was associated with a significant increase in risk of lung cancer (TT Versus CC, P=0.027, OR =1.67, 95% CI 1.06-2.63; recessive genetic model, *P*=0.32, OR=1.64, 95% CI 1.04-2.58). By contrast, a significant protective effect was found for colorectal cancer under the dominant genetic model (*P*=0.019, OR=0.84, 95% CI 0.72-0.97). However, no significant association was found in other tumor sites subgroups under all genetic models. These results are also listed in Table **3**.

In most of the available studies, there was no difference of MGMT Leu84Phe genotype/allele distribution among different ethnicities. We also performed stratified analysis by ethnicity (Caucasians, Asians, and mixed ethnicities), and by ethnicity and tumor site together (Table **4**). In subgroup meta-analysis by ethnicity, significant associations between TT and recessive genetic model and total cancer risk were found in the Caucasian population (TT versus CC, P=0.004, OR =1.32, 95% CI 1.10-1.61; recessive genetic model, *P*=0.002, OR=1.34, 95% CI 1.11-1.62) and in the mixed ethnicities population (TT versus CC, P=0.041, OR =1.27, 95% CI 1.01-1.60; recessive genetic model, *P*=0.037, OR=1.28, 95% CI 1.02-1.61). And, when those studies without consistency with HWE were excluded, a significant association was still found for the Caucasian population (TT versus CC, P=0.014, OR =1.29, 95% CI 1.05-1.59; recessive genetic model, *P*=0.009, OR=1.31, 95% CI 1.07-1.61). However, in the Asian subgroup and the mixed ethnicities subgroup, no significant association was observed for any genetic model. In the analysis stratified by ethnicity and tumor site (Table **4**), we found an increased risk only in the Caucasian subgroup for lung cancer (TT versus CC, P=0.035, OR =1.62, 95% CI 1.04-2.53; recessive genetic model, *P*=0.048, OR=1.57, 95% CI 1.01-2.45).

**Table 4:** Summary ORs (95% CI) for MGMT Leu84Phe variant categorized by ethnicity and ethnicity / tumor site under different genetic models.

MGMT Leu85Phe	N^#^	TT versus CC			TT+TC versus CC			TT versus TC + CC			T versus C	
					(dominant genetic model)			(recessive genetic model)				
Ethnicity		OR (95%CI)	*P*		OR (95%CI)	*P*		OR (95%CI)	*P*		OR (95%CI)	*P*
Caucasian	15	1.32 (1.10-1.61)	**0.004**		0.98 (0.90-1.06)^b^	0.560		1.34 (1.11-1.62)	**0.002**		1.00 (0.93-1.09)^b^	0.923
Caucasian in HWE	13	1.29 (1.05-1.59)	**0.014**		0.96 (0.88-1.06)^b^	0.407		1.31 (1.07-1.61)	**0.009**		0.99 (0.91-1.08)^b^	0.827
Asian	13	0.97 (0.58-1.61)	0.898		1.07 (0.88-1.31)^b^	0.485		0.94 (0.57-1.56)	0.805		1.03 (0.86-1.22)^b^	0.779
Asian in HWE	11	1.19 (0.68-2.09)	0.546		1.04 (0.83-1.30)^b^	0.724		1.15 (0.65-2.01)	0.633		1.02 (0.83-1.26)^b^	0.861
Mixed ethnicities	16	1.27 (1.01-1.60)	**0.041**		1.01 (0.91-1.13)^b^	0.813		1.28 (1.02-1.61)	**0.037**		1.04 (0.95-1.13)^b^	0.457
Mixed ethnicities in HWE	15	1.25 (0.99-1.58)	0.057		1.00 (0.90-1.12)^b^	0.997		1.26 (1.00-1.58)	0.052		1.08 (0.95-1.22)^b^	0.236
**Caucasian**												
Lung cancer	3	1.62 (1.04-2.53)	**0.035**		1.12 (0.96-1.31)	0.159		1.57 (1.01-2.45)	**0.048**		1.14 (0.99-1.31)	0.061
UADT SCC	3	0.88 (0.33-2.33)^b^	0.794		0.85 (0.66-1.08)^b^	0.182		0.92 (0.36-2.35)^b^	0.865		0.87 (0.66-1.14)^b^	0.312
**Asian**												
UADT SCC	3	0.94(01.15-5.84)	0.950		0.96(0.7101.30)	0.800		0.93 (0.15-5.76)	0.939		0.97 (0.73-1.28)	0.802
**Mixed ethnicities**												
Colorectal cancer	4	1.46 (0.89-2.38)	0.134		0.85 (0.72-1.01)	0.059		1.53 (0.94-2.50)	0.088		0.91 (0.79-1.06)	0.220

Bold indicates statistically significant *P* value

All summary ORs were calculated using fixed-effects models, unless stated otherwise

# Number of studies

b Random-effect models

UADT SCC − Upper Aerodigestive Tract Squamous Cell CarcinomaHWE − Hardy Weinberg Equilibrium

As shown in Table **3** and Table **4**, heterogeneity widely existed in the present meta-analysis under the dominant genetic mode and T versus C comparison but not under the homozygous comparison and recessive genetic model.

### Publication bias

**Figure 2:**
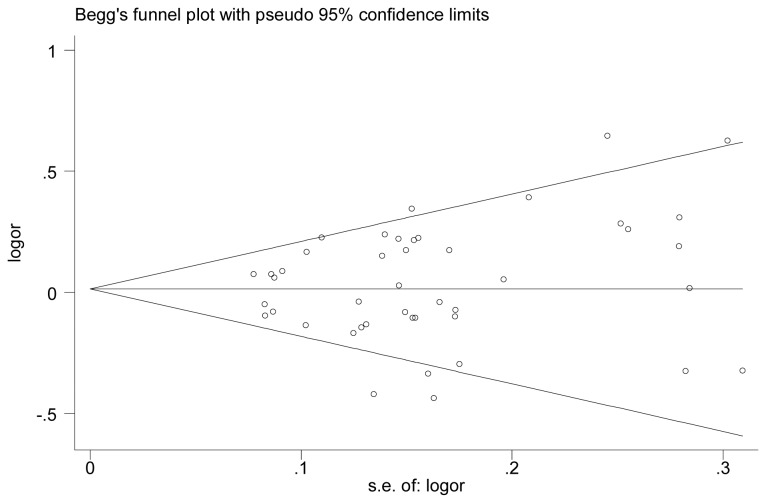
Begg’s funnel plot analysis to detect publication bias (MGMT : Leu84Phe T allele versus C allele). Each point represents a separate study for the indicated association. Logor represents natural logarithm of OR. Horizontal line represents the mean effects size.

Begg’s funnel plot and Egger’s test were utilized to evaluate the publication bias of the literature. As shown in Figure **2**, the contour-enhanced funnel plot for publication bias did not reveal any evidence of obvious asymmetry in allele contrast (T allele versus C allele), and, as expected, the Egger’s test did not provide any obvious evidence for bias (*t*=0.12, *P*=0.902).

## Discussion

This meta-analysis including a total of 18938 cancer patients and 28796 controls from 44 independent genetic studies implies that MGMT Leu84Phe polymorphism might contribute to the susceptibility of certain cancers

Although the global analysis indicated that the T variant allele might increase the risk of cancer, the subgroup meta-analysis showed significant association at only two tumor sites (colorectal cancer and lung cancer) and two ethnicity subgroups (Caucasian subgroup and mixed ethnicities subgroup). This phenomenon suggests that the MGMT Leu84Phe polymorphism may play differing roles in cancerogenesis at different sites or in different ethnicities because of variability in genetic backgrounds [62].

Since cancer is a complex disease, it is highly possible that any single genetic factor has only weak effects on an individual’s phenotype. It has been reported that the interaction of different combinations of polymorphisms in the same gene or between and among different genes might together have a pronounced effect on cancer risk [63,64,65]. Studies by Li et al. [66,67] have shown that MGMT is a transcriptional suppressor of ER-dependent signaling upon repair of the O^6^-methylguanine lesion and that the Lue84 and Ile143 residues lie in close proximity to three conserved leucines of the LXXLL ER-interacting helix. Therefore, it is possible that the ER-dependent signalling could be differentially mediated by the variant 84Phe and 143Val residues. Some studies [9,10,13,40,42,48,49,54] have tried to investigate the combined effects of Lue84Phe, Ile143Val, and other polymorphisms in MGMT on cancer risk. Because the available data were not compatible, we could not evaluate the combined effects of MGMT Leu84Phe and Ile143Val on cancer susceptibility in our meta-analysis.

It is well established that genetic factors may play an important role in the development of tumors. However, there is no doubt that environmental factors such as alcohol consumption, cigarette use, and aging also participate in tumorigenesis. Several studies [11,39,42] reported that heavy cigarette smoking could aggravate the effects of MGMT variants on cancer risk. However, Chae et al. [10] did not find the same results. Li et al. [40] found that both drinking and smoking enhance genetic variants’ effects on bladder cancer risk. It should be noted that alcohol consumption and cigarette use may play different roles at different tumor sites because of the different levels of alkylating agents and different tissue exposure concentrations. Unfortunately, owing to a lack of studies restricted to populations only exposed to alkylating agents, we could not obtain enough original data to further estimate the effects of the gene-environment interactions on cancer susceptibility.

We note several limitations in the present study. First, there was wide heterogeneity due to the nature of our meta-analysis, and the results should be interpreted with caution. Second, our results were based on unadjusted information, and the lack of original data limited estimation of the effect of confounding factors on cancer risk. Notably, confounding factors such as sex, age, alcohol drinking, smoking, and socioeconomic status may alter the association of genetic variants with cancer susceptibility. Third, the number of eligible studies in the subgroup analysis was limited. Subsequently, some subgroup meta-analysis might not have enough statistical power to accurately evaluate the association between the MGMT Leu84Phe polymorphism and cancer risk. More importantly, haplotype analysis has been regarded as a much better approach in genetic association research. However, since more detailed individual information on genotypes of the other polymorphisms of MGMT was unavailable, we were not able to conduct linkage disequilibrium and haplotype analysis in this study.

In conclusion, we observed several significant associations of the MGMT Leu84Phe polymorphism with cancer susceptibility. MGMT Leu84Phe variants may increase lung cancer risk, especially in Caucasians, but reduce colorectal cancer risk, indicating some differences among different tumor sites. In addition, MGMT Leu84Phe variants may increase cancer risk in Caucasians and in the mixed ethnicities group, which suggests an appreciable difference among different ethnic populations. Further well-designed study with greater sample size will be helpful in clarifying the haplotypes, gene–gene and gene–environment interactions on MGMT polymorphisms and tissue-specific cancer risk in ethnicity specific populations, and further mechanistic studies are warranted to elucidate the exact functional roles of MGMT variants.

## Supporting Information

Checklist S1 **PRISMA Checklist.**
(DOC)
