# Supplementary material for: MGMT Leu84Phe Polymorphism Contributes to Cancer Susceptibility: Evidence from 44 Case-Control Studies
Source: PLoS One. 2013 Sep 26;8(9):e75367. doi: 10.1371/journal.pone.0075367 (PMC3784571; doi:10.1371/journal.pone.0075367)
Supplement: Checklist S1 — PRISMA Checklist. (DOC) [file pone.0075367.s001.doc]

| **Section/topic** | **#** | **Checklist item** | **Reported in section** |
| --- | --- | --- | --- |
| **TITLE** | | |  |
| Title | 1 | MGMT Leu84Phe Polymorphism Contributes to Cancer Susceptibility: Evidence from 44 Case-Control Studies. | Title Page |
| **ABSTRACT** | | |  |
| Structured summary | 2 | **Background:** O^6^-methylguanine-DNA methyltransferase is one of the few proteins to directly remove alkylating agents in the human DNA direct reversal repair pathway. A large number of case-control studies have been conducted to explore the association between MGMT Leu84Phe polymorphism and cancer risk. However, the results were not consistent.  **Objectives:** to clarify the association between the Leu84Phe polymorphism and cancer risk.  **Data sources:** PubMed database (up to July 20, 2012);  study eligibility criteria: case-control studies and genotype frequencies in both cancer cases and controls were available.  **Study appraisal and synthesis methods**: a meta-analysis of 44 case-control studies was carried out with the software STATA v.10.0.  **Results:** Overall, significant association of the T allele with cancer susceptibility was verified with meta-analysis under a recessive genetic model (*P*<0.001, OR=1.30, 95%CI 1.24-1.50) and TT versus CC comparison (*P*=0.001, OR=1.29, 95% CI 1.12-1.50). In subgroup analysis, a significant increased risk was found for lung cancer (TT Versus CC, *P*=0.027, OR=1.67, 95% CI 1.06-2.63; recessive genetic model, *P*=0.32, OR=1.64, 95% CI 1.04-2.58), whereas risk of colorectal cancer was significantly low under a dominant genetic model (*P*=0.019, OR=0.84, 95% CI 0.72-0.97). Additionally, a significant association was found in the Caucasian population (TT versus CC, P=0.014, OR=1.29, 95% CI 1.05-1.59; recessive genetic model, P=0.009, OR=1.31, 95% CI 1.07-1.61), but not in the Asian population. An increased risk for lung cancer was also verified in the Caucasian population (TT versus CC, *P*=0.035, OR=1.62, 95% CI 1.04-2.53; recessive genetic model, *P*=0.048, OR=1.57, 95% CI 1.01-2.45).  **Limitations**: unadjusted information and the lack of original data limited estimation of the effect of confounding factors on cancer risk.  **Conclusions:** These results suggest that MGMT Leu84Phe polymorphism might contribute to the susceptibility of certain cancers.  Provide a structured summary including, as applicable: background; objectives; data sources; study eligibility criteria, participants, and interventions; study appraisal and synthesis methods; results; limitations; conclusions and implications of key findings; systematic review registration number. | Abstract |
| **INTRODUCTION** | | |  |
| Rationale | 3 | Numerous studies on the association of the *MGMT* Leu84Phe polymorphism with cancer risk have yeild inconsistent results and even partially contradictory conclusions. Since single studies may have been underpowered in clarifying this issue, an evidence-based quantitative meta-analyses is needed to address the controversy among literatures. | Introduction |
| Objectives | 4 | To address the controversy among literatures, in the present study we conducted an evidence-based quantitative meta-analyses of the association between the *MGMT* Leu84Phe polymorphism and susceptibility to cancer. | Introduction |
| **METHODS** | | |  |
| Protocol and registration | 5 | The study protocol was reviewed and approved by institutional review board of Yuhuangding Hospital. | Materials and Methods |
| Eligibility criteria | 6 | We carried out a computerized literature search without any restriction on language or publication year. Eligible studies should meet two criteria: (1) case-control studies; and (2) genotype frequencies in both cancer cases and controls were available. Exclusion criteria were as follows: (a) not relevant to MGMT Leu84Phe polymorphism; (b) not case-control study; (c) control population included malignant tumor cases; and (d) article was a review or duplication of previous publication. | *Identification and eligibility of relevant studies* |
| Information sources | 7 | PubMed database (up to July 20, 2012). | *Identification and eligibility of relevant studies* |
| Search | 8 | The key words used are as following: ‘MGMT,’ ‘polymorphism,’ and ‘cancer’.. | *Identification and eligibility of relevant studies* |
| Study selection | 9 | Eligible studies should meet two criteria: (1) case-control studies; and (2) genotype frequencies in both cancer cases and controls were available. | *Identification and eligibility of relevant studies* |
| Data collection process | 10 | The data was extracted by two investigators (Jun Liu and Fei Chen) from each article independently. Discrepancies were not solved until consensus was reached on every item. | *Data extraction* |
| Data items | 11 | From each study, the following data were collected: author’s name, year of publication, country of origin, racial descent, cancer type, source of the control population, genotyping methods, matched factors as well as adjusted factors, number of cases and controls, genotype frequencies for cases and controls, characteristics of cancer cases, and controls. | *Statistical analysis* |
| Risk of bias in individual studies | 12 | The Egger regression test and Begg-Mazumdar test were utilized to measure the potential publication bias. | *Statistical analysis* |
| Summary measures | 13 | Crude odds ratios (ORs) with 95% confidence intervals (CIs) were calculated to evaluate the strength of association between *MGMT*Leu84Phe polymorphism and cancer susceptibility. | *Statistical analysis* |
| Synthesis of results | 14 | In addition to overall meta-analysis, stratified analysis on ethnicity (Asians, Caucasians, and the other ethnicities group) and tumor site was also performed A ^2^–based Q-test was carried out to assess the heterogeneity of the ORs. | *Statistical analysis* |

Page 1 of 2

| **Section/topic** | **#** | **Checklist item** | **Reported in section** |
| --- | --- | --- | --- |
| Risk of bias across studies | 15 | The Egger regression test and Begg-Mazumdar test were utilized to measure the potential publication bias. | *Statistical analysis* |
| Additional analyses | 16 | In addition to overall meta-analysis, stratified analysis on ethnicity (Asians, Caucasians, and the other ethnicities group) and tumor site was also performed. | *Statistical analysis* |
| **RESULTS** | | |  |
| Study selection | 17 | The preliminary literature search yielded 46 articles that explored the association of MGMT polymorphisms with the susceptibility to different cancers. However, six articles irrelevant to MGMT Leu84Phe polymorphism and four articles without detailed MGMT Leu84Phe genotypes data were excluded. In addition, three articles were included by literature reading and manual searching. Therefore, 39 articles were identified and included in the final meta-analysis. | Results  *Characteristics of studies* |
| Study characteristics | 18 | See table 1. | *Characteristics of studies* |
| Risk of bias within studies | 19 | Begg’s funnel plot and Egger’s test were utilized to evaluate the publication bias of the literature. As shown in Figure.**2**, the contour-enhanced funnel plot for publication bias did not reveal any evidence of obvious asymmetry in allele contrast, and as expected, the Egger’s test did not provide any obvious evidence for bias (*t*=0.12, *P*=0.902). | *Quantitative synthesis* |
| Results of individual studies | 20 | See table 2. | Table 2 |
| Synthesis of results | 21 | See table 3. | *Quantitative synthesis* |
| Risk of bias across studies | 22 | See figure 2. | *Quantitative synthesis* |
| Additional analysis | 23 | See table 4. | *Quantitative synthesis* |
| **DISCUSSION** | | |  |
| Summary of evidence | 24 | Although the global analysis indicated that the T variant allele might increase the risk of cancer, the subgroup meta-analysis showed significant association at only two tumor sites (colorectal cancer and lung cancer) and two ethnicity subgroups (Caucasian subgroup and Other ethnicities subgroup). This phenomenon suggests that the *MGMT* Leu84Phe polymorphism may play differing roles in cancerogenesis at different sites or in different ethnicities because of variability in genetic backgrounds. | Discussion |
| Limitations | 25 | First, there was wide heterogeneity due to the nature of our meta-analysis, and the results should be interpreted with caution. Second, our results were based on unadjusted information, and the lack of original data limited estimation of the effect of confounding factors on cancer risk. Notably, confounding factors such as sex, age, alcohol drinking, smoking, and socioeconomic status may alter the association of genetic variants with cancer susceptibility. Third, the number of eligible studies in the subgroup analysis was limited. | Discussion |
| Conclusions | 26 | This meta-analysis including a total of 18938 cancer patients and 28796 controls from 44 independent genetic studies implies that MGMT Leu84Phe polymorphism might contribute to the susceptibility of certain cancers. | Discussion |
| **FUNDING** | | |  |
| Funding | 27 | Supported by Science and Technical Development Foundation of Shandong Province (2011YD18014), China; Doctoral Program of Shandong Province (2007BS03009), China; Science and Technical Development Foundation of Yantai (2008142-21), China. | Online submission system |

*From:*  Moher D, Liberati A, Tetzlaff J, Altman DG, The PRISMA Group (2009). Preferred Reporting Items for Systematic Reviews and Meta-Analyses: The PRISMA Statement. PLoS Med 6(6): e1000097. doi:10.1371/journal.pmed1000097

For more information, visit: **www.prisma-statement.org**.

Page 2 of 2
